# Assessing causal associations of hyperparathyroidism with blood counts and biochemical indicators: a Mendelian randomization study

**DOI:** 10.3389/fendo.2023.1295040

**Published:** 2023-12-11

**Authors:** Yan Jiang, Rumeng Chen, Shuling Xu, Yining Ding, Mengling Zhang, Meihua Bao, Binsheng He, Sen Li

**Affiliations:** ^1^School of Basic Medicine, Changsha Medical University, Changsha, China; ^2^The Hunan Provincial Key Laboratory of the TCM Agricultural Biogenomics, Changsha Medical University, Changsha, China; ^3^Hunan Key Laboratory Of The Research And Development Of Novel Pharmaceutical Preparations, School of Pharmaceutical Science, Changsha Medical University, Changsha, China; ^4^School of Life Sciences, Beijing University of Chinese Medicine, Beijing, China; ^5^School of Stomatology, Changsha Medical University, Changsha, China

**Keywords:** hyperparathyroidism, blood counts, biochemical indicators, Mendelian randomization, causal association

## Abstract

**Background:**

The existing literature on the relationship of hyperparathyroidism with both blood counts and biochemical indicators primarily comprises observational studies, which have produced inconsistent findings. This study aimed to evaluate the causal relationship between hyperparathyroidism and blood counts and biochemical indicators.

**Methods:**

Mendelian randomization (MR) analyses were conducted to investigate the associations between hyperparathyroidism and the identified 55 blood counts and biochemical indicators. The genome-wide association study (GWAS) for hyperparathyroidism data was obtained from FinnGen, while the GWASs for the blood counts and biochemical indicators were sourced from the UK Biobank (UKBB).

**Results:**

The MR analysis using the inverse-variance weighted (IVW) method revealed potential causality between genetically predicted hyperparathyroidism and seven out of 55 blood counts and biochemical indicators. These markers include “Platelet count” (Beta = -0.041; 95% CI: -0.066, -0.016; *p* = 0.001), “Platelet distribution width (PDW)” (Beta = 0.031; 95% CI: 0.006, 0.056; *p* = 0.016), “Mean platelet volume (MPV)” (Beta = 0.043; 95% CI: 0.010, 0.076; *p* = 0.011), “Vitamin D” (Beta = -0.038; 95% CI: -0.063, -0.013; *p* = 0.003), “Calcium (Ca^2+^)” (Beta = 0.266; 95% CI: 0.022, 0.509; *p* = 0.033), “Phosphate” (Beta = -0.114; 95% CI: -0.214, -0.014; *p* = 0.025), and “Alkaline phosphatase (ALP)” (Beta = 0.030; 95% CI: 0.010, 0.049; *p* = 0.003).

**Conclusion:**

The findings of our study revealed a suggestive causal relationship between hyperparathyroidism and blood cell count as well as biochemical markers. This presents a novel perspective for further investigating the etiology and pathological mechanisms underlying hyperparathyroidism.

## Introduction

Hyperparathyroidism is caused by various factors that stimulate the excessive secretion of the parathyroid hormone (PTH) from parathyroid cells. This hormonal imbalance disrupts calcium (Ca^2+^) and phosphorus metabolism, resulting in symptoms such as bone pain, fractures, urinary stones, and other conditions that significantly impact patients’ quality of life (1, 2). The diagnosis of hyperparathyroidism depends on auxiliary examinations such as biochemical and ultrasound examinations. In recent years, there has been an increase in the prevalence of hyperparathyroidism. This can be attributed partially to regular monitoring of laboratory serum Ca^2+^ levels and the incorporation of serum Ca^2+^ and PTH testing into osteoporosis screening protocols (3).

Primary hyperparathyroidism (PHPT) is the most prevalent variant of hyperparathyroidism. PHPT ranks third among endocrine disorders, following diabetes mellitus and thyroid disease (4). In total, 85% of the subjects exhibited PHPT due to a parathyroid adenoma (5). Tumor cells secrete bone marrow growth factors, leading to an increase in neutrophil production, which can be related to inflammation (6, 7). Neutrophils, in turn, secrete cytokines, and cytokines can regulate the proliferation and invasion of tumor cells (8–10). Additionally, tumor cells secrete cytokines and growth factors that induce differentiation of megakaryocytes, leading to enhanced platelet production (8). High levels of PTH in hyperparathyroidism suppress hematopoiesis (11). Studies have also revealed the significance of lymphocytes as anti-tumor cells. A change in lymphocyte number has been observed in tumor development, and their decrease may impact the response to tumor cells (12, 13). Therefore, blood cells may contribute to the pathogenesis of PHPT. There is limited research on blood counts and hyperparathyroidism.

Blood counts and biochemical indicators are widely utilized as biomarkers in the literature due to their ease of use and high patient acceptance (14, 15). An increasing number of studies have identified an association between PTH and blood counts as well as biochemical indicators (16–18). However, discrepancies arise in the conclusions derived from multiple studies investigating the connection between PTH and blood counts as well as biochemical indicators. For instance, Emam et al. demonstrated a substantial correlation between serum PTH and C-reactive protein (19). Conversely, another study presented contradictory outcomes (20). Hence, additional investigations into the causal association between PTH and blood counts as well as biochemical indicators are necessary.

Mendelian Randomization (MR) analysis represents an approach that utilizes genetic variants linked to the exposures as instrumental variables (IVs). Its primary aim is to evaluate possible causal connections between the exposure and outcome (21, 22). MR analysis relies on Mendel’s second law, which asserts that alleles adhere to a principle of random allocation. This characteristic helps mitigate biases stemming from confounding factors and reverse causation, regardless of disease status involvement. This study aimed to employ MR design to examine the presence of a causal relationship between hyperparathyroidism and 55 blood counts and biochemical indicators.

## Methods

### Study design

Single nucleotide polymorphisms (SNPs) are widely utilized as IVs in MR, a method that estimates the causal relationships between traits and diseases. In this study, we conducted MR analyses using data from genome-wide association studies (GWAS) datasets to examine the association between hyperparathyroidism as exposure and 55 blood counts and biochemical indicators as outcomes.

### Data sources

For our MR analysis, we utilized diverse sets of publicly accessible summary data from GWAS. To meet the criteria of a two-sample MR design, we sourced the exposure and outcome variables from distinct European populations. We acquired hyperparathyroidism GWAS data from FinnGen, which is a biomedical research initiative in Finland (23). Moreover, we retrieved summary statistics regarding 55 outcomes from the UK Biobank (UKBB). Further information can be found in **Supplementary Table 1**.

### Statistical method

In our MR analysis, we employed specific criteria to select IVs: (1) significant genomic-level association between IVs and exposure (*p*< 5.00×10^-8^), (2) independent selection of IVs achieved by clumping within a 10 Mb window and low linkage disequilibrium (R^2^ < 0.001), and (3) ensuring that minor allele frequency (MAF) was greater than 0.01. The F-statistics are used to assess the strength of IVs, with values exceeding 10 indicating a reduced risk of weak instrument bias (24).

In our MR analysis, we primarily employed the inverse-variance weighted (IVW) method as our main strategy. This approach used inverse-variance based weighting, and constrained the regression line intercept to cross zero (25). Furthermore, we employed two additional methods – weighted median (WM) and MR-Egger – as sensitivity analyses. The WM method maintains its unbiasedness as long as less than 50% of the weight is derived from unreliable instruments. MR-Egger does not make assumptions about the regression line intersecting at zero and can be used to estimate potential horizontal pleiotropy (26). We utilized data from MR-PRESSO analysis to identify and remove potential outliers. Heterogeneity was evaluated using the Cochrane Q value. Additionally, we assessed the robustness of our findings by employing a leave-one-out strategy that examined how each IV influenced the causal associations. For causal estimations, we calculated beta coefficients along with their corresponding 95% confidence intervals (CIs). To account for multiple comparisons, we applied a false discovery rate (FDR) threshold of 5%. All MR analyses were performed using the Two-Sample MR package in R.

## Results

In total, 8 distinct SNPs were identified as the genetic IVs for hyperparathyroidism in our MR analyses based on the inclusion and exclusion criteria, and the conditional F-statistics for the IVs varied from 29.93 to 86.81, showing good instrument strength (**Supplementary Table 2**).

**Figure 1:**
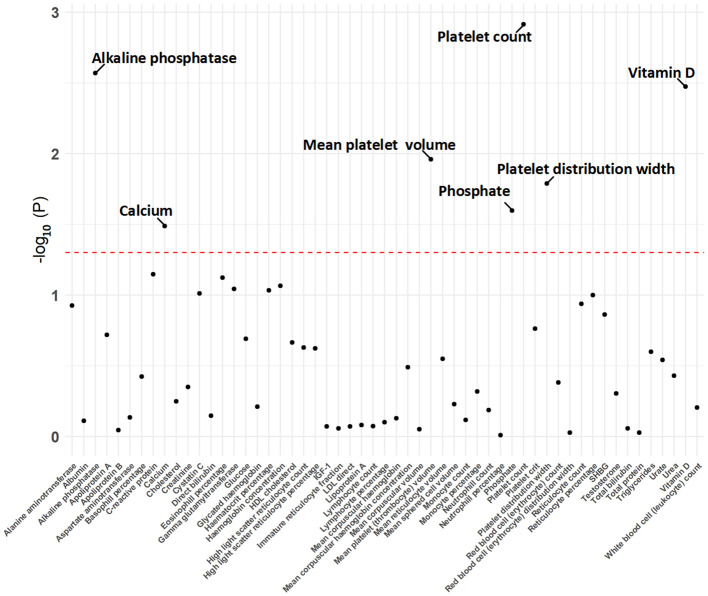
The *p*-value distribution of associations between hyperparathyroidism and 55 blood counts and biochemical indicators in the Mendelian randomization analysis. The dashed line represents the threshold of suggestive level of significance, set at *p*= 0.05.

**Figure 2:**
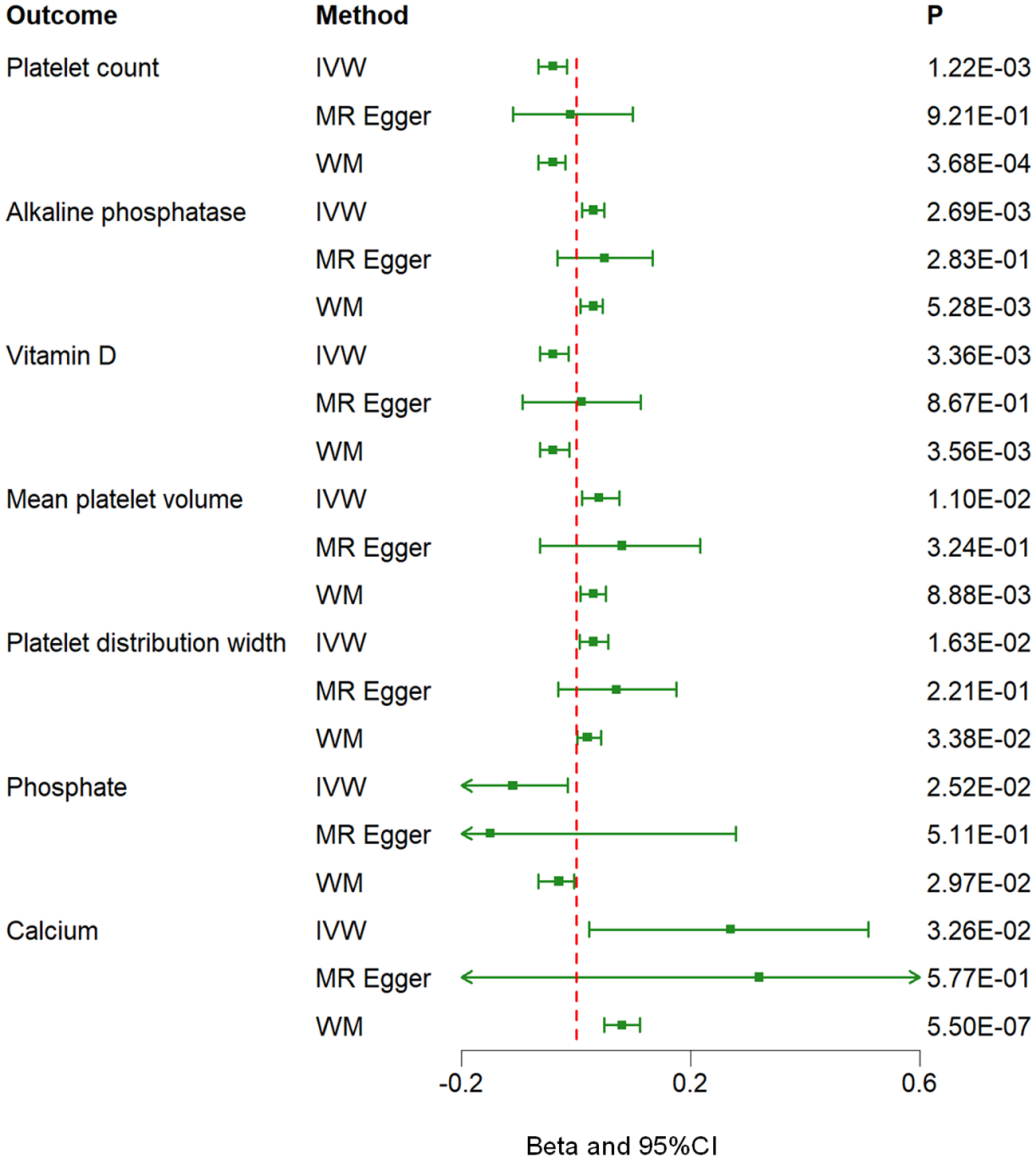
Associations between genetically predicted hyperparathyroidism and 7 blood counts and biochemical indicators examined by three MR methods. MR, Mendelian randomization; IVW, inverse-variance weighted; WM, weighted median; CI, confidence interval.

**Figure 3:**
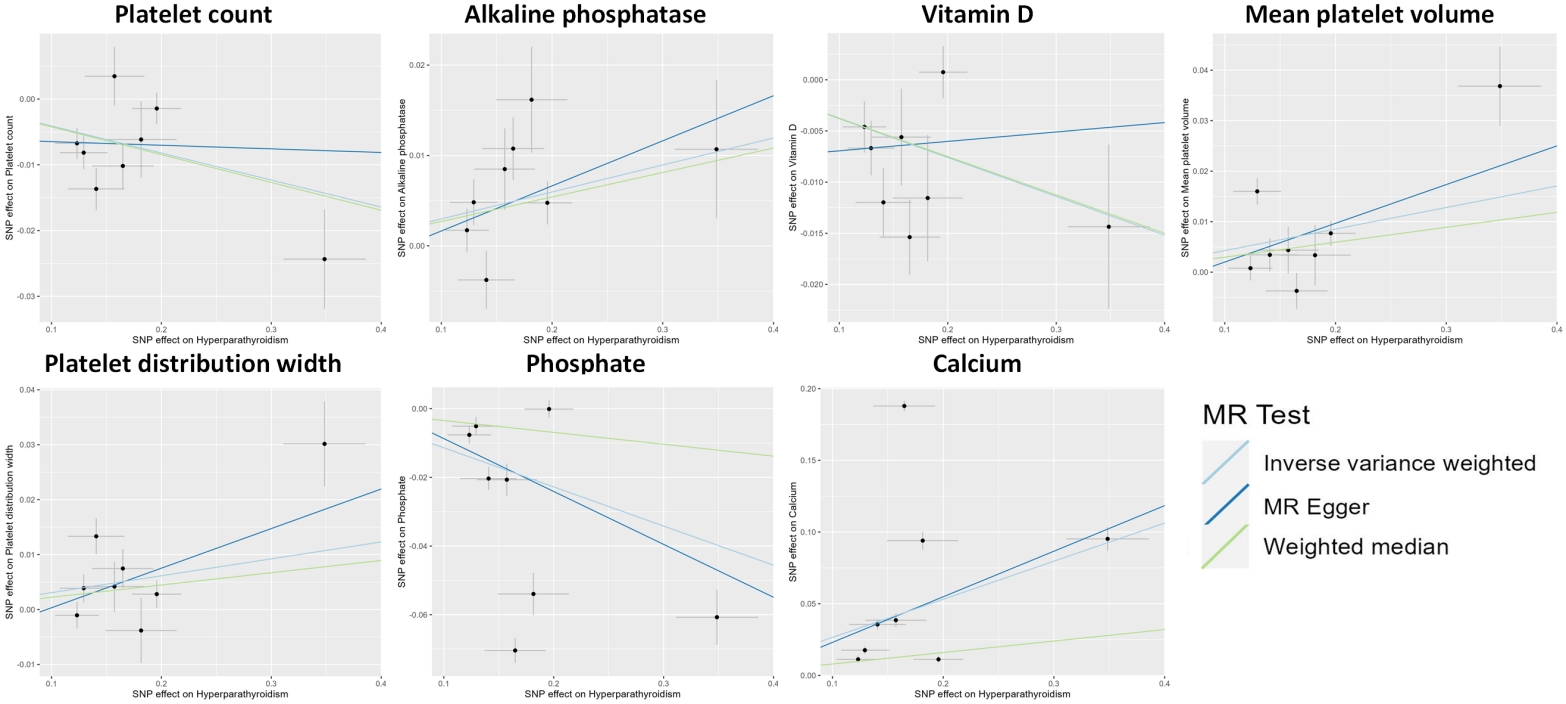
Scatter plot showing the causal effects of hyperparathyroidism on 7 blood counts and biochemical indicators. SNP, single nucleotide polymorphism.

The MR analysis using the IVW method revealed that genetically predicted hyperparathyroidism showed potential causality with seven out of 55 blood counts and biochemical indicators, including “Platelet count” (Beta = -0.041; 95% CI: -0.066, -0.016; *p* = 0.001), “Platelet distribution width (PDW)” (Beta = 0.031; 95% CI: 0.006, 0.056; *p* = 0.016), “Mean platelet (thrombocyte) volume (MPV)” (Beta = 0.043; 95% CI: 0.010, 0.076; *p* = 0.011), “Vitamin D” (Beta = -0.038; 95% CI: -0.063, -0.013; *p* = 0.003), “Ca^2+^” (Beta = 0.266; 95% CI: 0.022, 0.509; *p* = 0.033), “Phosphate” (Beta = -0.114; 95% CI: -0.214, -0.014; *p* = 0.025), and “Alkaline phosphatase (ALP)” (Beta = 0.030; 95% CI: 0.010, 0.049; *p* = 0.003) (**Figures 1**, **2**; **Supplementary Table 3**). Although none of these associations survived multiple comparisons with 5% FDR, suggestive level of significance (*p*<0.05) was observed. Using the MR-Egger and WM approaches, the relationships between hyperparathyroidism and 7 of 55 blood counts and biochemical indicators had the same direction except for Vitamin D (**Figure 2**; **Supplementary Table 3**). **Figure 3** displays the scatter plot illustrating the causal relationships between hyperparathyroidism and 7 blood counts and biochemical indicators.

**Figure 4:**
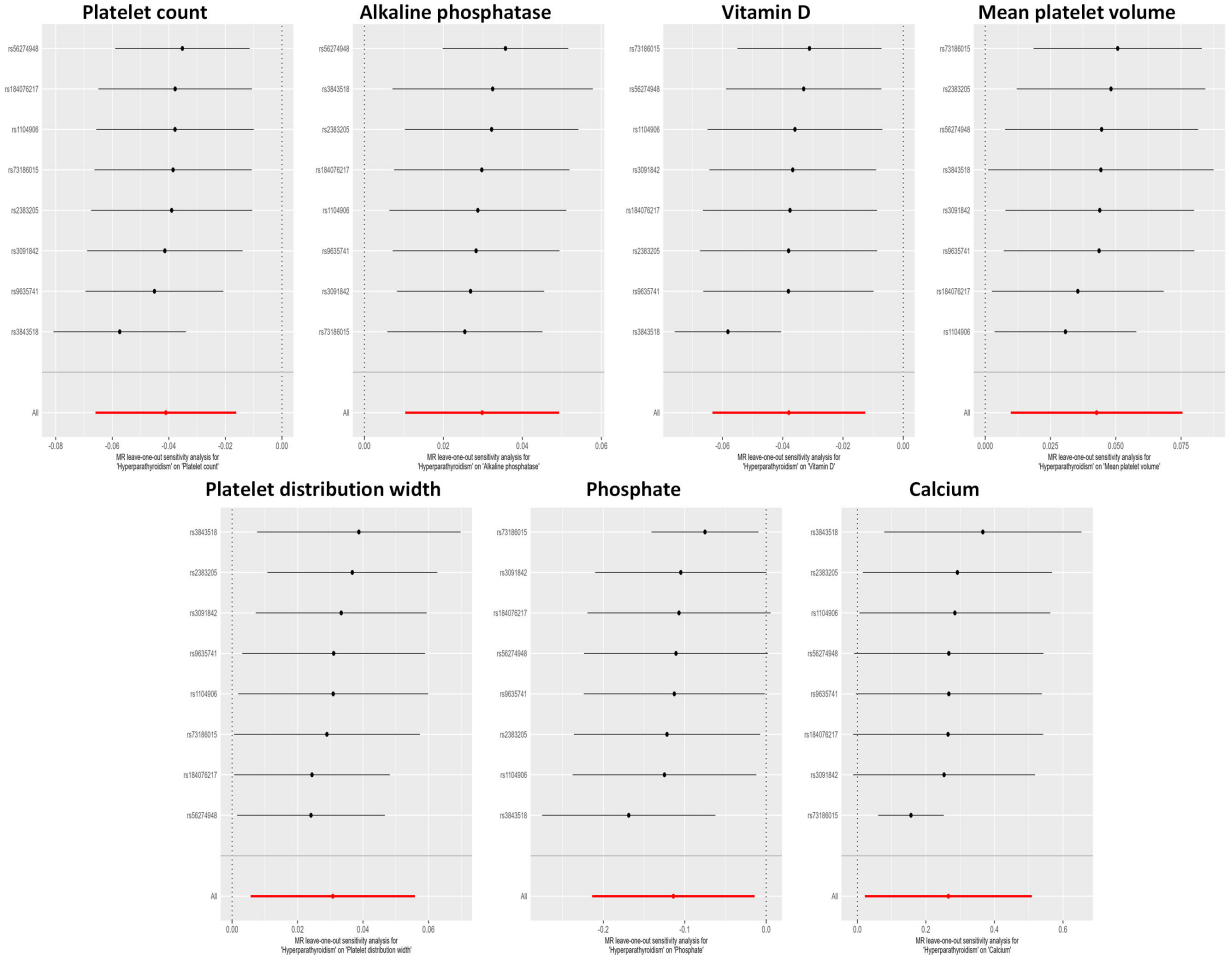
Leave-one-out sensitivity analysis examining the causal estimates of hyperparathyroidism on 7 blood counts and biochemical indicators by the IVW method after excluding a specific SNP from the analysis. The red line represents the IVW estimate of all SNPs on each outcome. MR, Mendelian randomization; SNP, single nucleotide polymorphism; IVW, inverse-variance weighted.

The potential heterogeneity was also analyzed (**Supplementary Figure 1**; **Supplementary Table 4**). The findings depicted in **Figure 4** indicate that during a leave-one-out analysis, most individual SNPs do not exert a substantial influence on the result. Nevertheless, it was observed that the removal of certain SNPs in the analyses of Ca^2+^ and phosphate had an impact on the overall results. Notably, there was no change in the direction of the results after removing these specific SNPs. The causality analysis did not show significant evidence of horizontal pleiotropy based on the examination of the MR-Egger method’s intercept term (**Supplementary Table 5**). The MR-PRESSO analysis identified outliers in several results, and all the results, except the association of hyperparathyroidism with platelet distribution width, remained significant at suggestive level of evidence after outlier correction (**Supplementary Table 6**).

Additionally, we investigated reverse causality by considering seven blood cell counts and biochemical indicators as potential exposures, with hyperparathyroidism as the outcome. The results indicated a suggestive causal relationship between ALP, Ca^2+^, phosphate, and hyperparathyroidism (**Supplementary Table 7**).

## Discussion

This study employed the MR approach to examine the association between hyperparathyroidism and 55 blood counts and biochemical indicators. The results revealed a suggestive causal relationship between hyperparathyroidism and seven blood counts and biochemical indicators (*p*<0.05). More specifically, it demonstrated a suggestive negative causal correlation of hyperparathyroidism with platelet count, vitamin D, and phosphate levels (*p*<0.05). Furthermore, there were suggestive positive causal associations observed between hyperparathyroidism and ALP along with MPV, PDW, and Ca^2+^ concentration (*p*<0.05).

### Calcium and phosphate

This study uncovered a suggestive positive causal association between hyperparathyroidism and serum Ca^2+^ (*p*<0.05), as well as a suggestive negative causal association with phosphate (*p*<0.05), which aligns with prior research findings. Previous studies have documented that PHPT is characterized by hypercalcemia and elevated or abnormal PTH levels. Additionally, serum phosphate levels in patients with PHPT are commonly low or abnormal and demonstrate an inverse correlation with PTH levels (27). The underlying mechanism may be the activation of the calcium-sensitive receptor (CaSR) found on cell membranes. Ca^2+^ is the most sensitive ligand for the membrane-associated CaSR. Even minor changes in Ca^2+^ can activate the membrane-associated CaSR. CaSR activation not only inhibits the secretion of PTH and the expression of PTH genes but also suppresses the proliferation of parathyroid cells (3). Phosphate directly stimulates parathyroid hormone secretion by acting on CaSR (28). Additionally, cinacalcet enhances the responsiveness of the CaSR to extracellular Ca^2+^, resulting in decreased PTH levels and subsequently lowering serum Ca^2+^ concentrations (29).

### Vitamin D

This study discovered a suggestive negative causality between hyperparathyroidism and vitamin D (*p*<0.05). This finding is corroborated by previous research. A cross-sectional study revealed a significant inverse association between serum 25-hydroxyvitamin D (25-(OH)D) levels and PTH concentrations (30). Moreover, in an additional randomized controlled trial, PTH levels exhibited a decrease following vitamin D treatment in patients with PHPT (31). The potential mechanism of action is the enhancement of CaSR expression by vitamin D in parathyroid cells (32). CaSR is a G protein-coupled receptor situated on the parathyroid gland and plays a crucial role as a negative regulator of PTH secretion through its ability to sense changes in serum Ca^2+^ levels (33). This regulation is vital for inhibiting excessive hyperplasia of the parathyroid glands. In summary, upregulation of CaSR leads to increased sensitivity of PTH to extracellular Ca²^+^, ultimately resulting in decreased secretion of PTH.

### ALP

This study identified a suggestive positive causal relationship between hyperparathyroidism and ALP levels, which is consistent with previous relevant studies. ALP is a glycoprotein that catalyzes the hydrolysis of phosphomonoesters (34). ALP is classified into different types based on its distribution: placental ALP, germ cell ALP, intestinal ALP, and bone ALP (BALP) (35). The soluble BALP has been proposed as a biomarker for bone formation (35). A significant inverse relationship between PTH levels and ALP activity was discovered in previous research in foals (36). However, our findings contradict this, possibly due to inter-species variations. In a cohort study, it was discovered that elevated serum intact parathyroid hormone (iPTH) levels were an independent predictor of increased ALP levels, and elevated ALP levels were an independent predictor of high iPTH levels (37). Furthermore, an observational study revealed a positive linear correlation between elevated PTH levels and ALP levels. Moreover, ALP was identified as a strong and statistically significant predictor of serum iPTH levels (38). The underlying mechanism may be associated with the receptor activator of nuclear factor kappa-B ligand (RANKL). Elevated serum ALP levels lead to the binding of serum iPTH to osteoblasts through the PTH receptor-1, which in turn stimulates an increase in the expression of the RANKL receptor activator. Additionally, the binding of RANKL to its receptor triggers the fusion of osteoclast precursors, resulting in the formation of new osteoclasts (38).

### Platelet

MPV, PDW, and platelet count are all parameters used to assess platelet activity (39). This study establishes an association between hyperparathyroidism and platelets. The study demonstrated a suggestive negative causal relationship between hyperparathyroidism and platelet count, while also indicating a suggestive positive causal relationship between hyperparathyroidism and both MPV and PDW (*p*<0.05). In contrast to our study findings, a prior retrospective study failed to identify a significant association between PTH and platelet count (18). Being an observational design, it is unable to fully account for potential confounding variables. MPV serves as an important parameter indicating the average size of platelets. It reflects their rate of generation and stimulation while functioning as a bioactive marker (40). In a recent study conducted by Yilmaz et al., it was found that individuals with PHPT exhibited increased platelet activation (41). Additionally, Irkorucu et al.’s research demonstrated a significant association between MPV levels and parathyroid adenomas, often coexisting with hyperthyroidism (40). Moreover, Yilmaz’s investigation reported positive correlations between MPV values and PTH levels (*r* = 0.888; *p* < 0.0001) (41). PDW, an indicator of platelet activation, is reflective of the variation in the size of circulating platelets (39). PDW is obtained by analyzing the size distribution curve of platelets, with the width at the 20% level being defined as PDW (42). A retrospective study of patients with PHPT revealed a significant elevation in the preoperative PDW value when compared to both the postoperative period and control groups (43). These outcomes are in line with what our study revealed. The association between hyperparathyroidism and platelet count can be explained by the potential mechanism of myelofibrosis induced by elevated levels of PTH. Elevated PTH levels can directly stimulate bone marrow fibroblasts or indirectly promote bone fibrosis by activating osteoclasts and causing bone resorption (44). This process leads to the release of cytokines, such as interleukin 6 and tumor necrosis factor α, which can in turn impair the production and effectiveness of thrombopoietin. Moreover, myelofibrosis in the bone marrow can result in a decrease in total blood cell count (44). Given the limited number of existing studies, further investigation is necessary to fully understand the underlying mechanism connecting hyperparathyroidism with MPV and PDW.

### Strengths and limitations

This study exhibits several noteworthy strengths. Firstly, it utilized a two-sample MR strategy to mitigate the impact of confounding factors and reverse causality on the outcome. Secondly, it stands as the first extensive investigation to date on the causal relationship between hyperparathyroidism and a diverse range of blood counts and biochemical indicators. Lastly, robust findings were achieved through the utilization of recent and reliable GWAS summary statistics, coupled with the implementation of multiple sensitivity analyses.

The study’s limitations should also be acknowledged. Firstly, the MR analysis did not consider the influence of epigenetic factors, specifically methylation, on the results. Furthermore, the interaction between genes and environmental exposures may also affect the association between hyperparathyroidism and blood counts as well as biochemical indicators, potentially influencing the underlying mechanisms of this association across different populations. Secondly, the populations included in this study were exclusively from Europe, which raises concerns about the potential for spurious associations if the results are extrapolated directly to other ethnic groups. This is due to variations in trait distribution and allele frequencies among populations. To enhance the generalizability of the findings, future research should investigate potential causal relationships in more ethnically diverse groups. Thirdly, the possible premature death of some patients with hyperparathyroidism, resulting from conditions such as cancer or uremia, may have made the genotyping of SNP in these individuals impossible. Consequently, the data in the GWAS became incomplete, introducing selection bias. Fourthly, MR analysis assumes a linear correlation between exposure and outcome. However, the relationship between exposure and outcome can be non-linear, as seen in the inverse relationship between 25 (OH) D concentration and PTH (45). This non-linearity can introduce bias in the results of MR analysis. Fifthly, although the study has identified a potential causal relationship between hyperparathyroidism and the mentioned blood counts and biochemical indicators, future validation through cohort studies or clinical trials is necessary. Lastly, it is important to note that this study does not provide definitive recommendations, but rather presents certain trends. The study identified suggestive causation with a significance level of *p*<0.05; however, these findings did not remain significant after adjusting for multiple comparisons. Validation of the findings through future studies with larger sample sizes is warranted.

## Conclusion

The IVW analysis demonstrated a suggestive negative causal association between hyperparathyroidism and platelet count, vitamin D levels, and phosphate levels with a suggestive level of evidence. Furthermore, a suggestive positive causal relationship was observed between hyperparathyroidism and ALP, MPV, PDW, and Ca^2+^ concentrations. Our study offers a new direction for investigating the etiology and pathological mechanisms underlying hyperparathyroidism. Furthermore, additional research is warranted to delve deeper into this topic.

## Data availability statement

This study analyzed the exposure data acquired from FinnGen and the outcome data sourced from the UKBB. Both datasets are publicly accessible and can be found on the IEU Open GWAS Project website (https://gwas.mrcieu.ac.uk/).

## Ethics statement

The GWASs included in this work were approved by their relevant review board, and informed consent were given by all participants.

## Author contributions

YJ: Writing – original draft. RC: Writing – original draft. SX: Writing – original draft. YD: Writing – original draft. MZ: Writing – original draft. MB: Writing – review & editing. BH: Writing – review & editing. SL: Writing – review & editing.

## Supplementary material

Supplementary Table 1 Information of GWAS summary datasets used in MR analyses.

Supplementary Table 2 The F-statistics of IVs.

Supplementary Table 3 MR analysis results.

Supplementary Table 4 Heterogeneity test results of associations with suggestive significant level.

Supplementary Table 5 The pleiotropy results.

Supplementary Table 6 MR-PRESSO analyses for the results with identified outliers.

Supplementary Table 7 Bidirectional MR analyses results.

Supplementary Figure 1 Funnel plot indicating the causal associations of hyperparathyroidism on 7 blood counts and biochemical indicators, with each SNP acting as an IV. SNP: single nucleotide polymorphism; IV: instrumental variable; SE: standard error.

## References

[1] Fraser WD. Hyperparathyroidism. Lancet (2009) 374(9684):145–58. doi: 10.1016/S0140-6736(09)60507-9 19595349

[2] Chen Y Xiang J Wang Z Xiao Y Zhang D Chen X. Associations of bone mineral density with lean mass, fat mass, and dietary patterns in postmenopausal chinese women: A 2-year prospective study. PloS One (2015) 10(9):e0137097. doi: 10.1371/journal.pone.0137097 26335921 PMC4559415

[3] Minisola S Arnold A Belaya Z Brandi ML Clarke BL Hannan FM. Epidemiology, pathophysiology, and genetics of primary hyperparathyroidism. J Bone Miner Res (2022) 37(11):2315–29. doi: 10.1002/jbmr.4665 PMC10092691 36245271

[4] Younes NA Shafagoj Y Khatib F Ababneh M. Laboratory screening for hyperparathyroidism. Clin Chim Acta (2005) 353(1-2):1–12. doi: 10.1016/j.cccn.2004.10.003 15698586

[5] Koufakis T Antonopoulou V Grammatiki M Karras SN Ajjan R Zebekakis P. The relationship between primary hyperparathyroidism and thrombotic events: report of three cases and a review of potential mechanisms. Int J hematology-oncology Stem Cell Res (2018) 12(3):175–80. Available at: https://www.ncbi.nlm.nih.gov/pmc/articles/PMC6305263/. PMC6305263 30595818

[6] Zhu LM Zeng D Lei XC Huang J Deng YF Ji YB. KLF2 regulates neutrophil migration by modulating CXCR1 and CXCR2 in asthma. Biochim Biophys Acta Mol Basis Dis (2020) 1866(12):165920. doi: 10.1016/j.bbadis.2020.165920 32800946

[7] Luo W Tian L Tan B Shen Z Xiao M Wu S. Update: innate lymphoid cells in inflammatory bowel disease. Dig Dis Sci (2022) 67(1):56–66. doi: 10.1007/s10620-021-06831-8 33609209

[8] Marques P de Vries F Dekkers OM Korbonits M Biermasz NR Pereira AM. Serum inflammation-based scores in endocrine tumors. J Clin Endocrinol Metab (2021) 106(10):e3796–e819. doi: 10.1210/clinem/dgab238 PMC8475227 33837783

[9] Chen S Chen Y Yu L Hu X. Overexpression of SOCS4 inhibits proliferation and migration of cervical cancer cells by regulating JAK1/STAT3 signaling pathway. Eur J Gynaecological Oncol (2021) 42(3):554–60. doi: 10.31083/j.ejgo.2021.03.2416

[10] Shen F Long D Yu T Chen X Liao Y Wu Y. Vinblastine differs from Taxol as it inhibits the Malignant phenotypes of NSCLC cells by increasing the phosphorylation of Op18/stathmin. Oncol Rep (2017) 37(4):2481–9. doi: 10.3892/or.2017.5469 28259950

[11] Kotzmann H Abela C Heindl J Clodi M Riedl M Barnas U. Effect of successful parathyroidectomy on hematopoietic progenitor cells and parameters of red blood cells in patients with primary hyperparathyroidism. Hormone Metab Res = Hormon- und Stoffwechselforschung = Hormones metabolisme (1997) 29(8):387–92. doi: 10.1055/s-2007-979061 9288576

[12] Tang H Lu W Li B Li C Xu Y Dong J. Prognostic significance of neutrophil-to-lymphocyte ratio in biliary tract cancers: a systematic review and meta-analysis. Oncotarget (2017) 8(22):36857–68. doi: 10.18632/oncotarget.16143 PMC5482704 28415734

[13] Zhao Y Chen S Shen F Long D Yu T Wu M. *In vitro* neutralization of autocrine IL−10 affects Op18/stathmin signaling in non−small cell lung cancer cells. Oncol Rep (2019) 41(1):501–11. doi: 10.3892/or.2018.6795 30320402

[14] Chen L Liu W Lai S Li Y Wang X Zhang H. Insulin resistance, serum visfatin, and adiponectin levels are associated with metabolic disorders in chronic hepatitis C virus-infected patients. Eur J Gastroenterol Hepatol (2013) 25(8):935–41. doi: 10.1097/MEG.0b013e32835fa988 23470357

[15] Tang L Wang Y Chen BF. A variant within intron 1 of the PTPN22 gene decreases the genetic susceptibility of ankylosing spondylitis in a central south Chinese Han population. Scand J Rheumatol (2014) 43(5):380–4. doi: 10.3109/03009742.2014.899390 24749936

[16] Alemzadeh R Kichler J. Parathyroid hormone is associated with biomarkers of insulin resistance and inflammation, independent of vitamin D status, in obese adolescents. Metab Syndr Relat Disord (2012) 10(6):422–9. doi: 10.1089/met.2012.0056 22849753

[17] Cheng S-P Liu C-L Liu T-P Hsu Y-C Lee J-J. Association between parathyroid hormone levels and inflammatory markers among US adults. Mediators Inflamm (2014) 2014:709024. doi: 10.1155/2014/709024 24782595 PMC3980926

[18] Lam HB Yang PS Chien MN Lee JJ Chao LF Cheng SP. Association between neutrophil-to-lymphocyte ratio and parathyroid hormone in patients with primary hyperparathyroidism. Arch Med Sci AMS (2019) 15(4):880–6. doi: 10.5114/aoms.2018.74758 PMC6657247 31360183

[19] Emam AA Mousa SG Ahmed KY Al-Azab AA. Inflammatory biomarkers in patients with asymptomatic primary hyperparathyroidism. Med Princ Pract (2012) 21(3):249–53. doi: 10.1159/000334588 22179481

[20] Chertok-Shacham E Ishay A Lavi I Luboshitzky R. Biomarkers of hypercoagulability and inflammation in primary hyperparathyroidism. Med Sci Monit (2008) 14(12):CR628–CR32. Available at: https://medscimonit.com/abstract/index/idArt/869485. 19043371

[21] Burgess S Butterworth A Malarstig A Thompson SG. Use of Mendelian randomisation to assess potential benefit of clinical intervention. BMJ (2012) 345:e7325. doi: 10.1136/bmj.e7325 23131671

[22] Chen R Xu S Ding Y Li L Huang C Bao M. Dissecting causal associations of type 2 diabetes with 111 types of ocular conditions: a Mendelian randomization study. Front Endocrinol (Lausanne) (2023) 14. doi: 10.3389/fendo.2023.1307468 PMC10703475 38075077

[23] Kurki MI Karjalainen J Palta P Sipilä TP Kristiansson K Donner KM. FinnGen provides genetic insights from a well-phenotyped isolated population. Nature. (2023) 613(7944):508–18. doi: 10.1038/s41586-022-05473-8 PMC9849126 36653562

[24] Burgess S Thompson SG. Avoiding bias from weak instruments in Mendelian randomization studies. Int J Epidemiol. (2011) 40(3):755–64. doi: 10.1093/ije/dyr036 21414999

[25] Hemani G Bowden J Davey Smith G. Evaluating the potential role of pleiotropy in Mendelian randomization studies. Hum Mol Genet (2018) 27(R2):R195–208. doi: 10.1093/hmg/ddy163 PMC6061876 29771313

[26] Bowden J Davey Smith G Burgess S. Mendelian randomization with invalid instruments: effect estimation and bias detection through Egger regression. Int J Epidemiol. (2015) 44(2):512–25. doi: 10.1093/ije/dyv080 PMC4469799 26050253

[27] Düğer H Bostan H Gül Ü Uçan B Hepşen S Sakız D. The importance of hypophosphatemia in the clinical management of primary hyperparathyroidism. J Endocrinol Invest. (2023) 46(9):1935–40. doi: 10.1007/s40618-023-02064-w 36929458

[28] Centeno PP Herberger A Mun HC Tu C Nemeth EF Chang W. Phosphate acts directly on the calcium-sensing receptor to stimulate parathyroid hormone secretion. Nat Commun (2019) 10(1):4693. doi: 10.1038/s41467-019-12399-9 31619668 PMC6795806

[29] Khan A Bilezikian J Bone H Gurevich A Lakatos P Misiorowski W. Cinacalcet normalizes serum calcium in a double-blind randomized, placebo-controlled study in patients with primary hyperparathyroidism with contraindications to surgery. Eur J Endocrinology (2015) 172(5):527–35. doi: 10.1530/EJE-14-0877 PMC5729741 25637076

[30] Walker MD Silverberg SJ. Primary hyperparathyroidism. Nat Rev Endocrinology (2018) 14(2):115–25. doi: 10.1038/nrendo.2017.104 PMC6037987 28885621

[31] Rolighed L Rejnmark L Sikjaer T Heickendorff L Vestergaard P Mosekilde L. Vitamin D treatment in primary hyperparathyroidism: a randomized placebo controlled trial. J Clin Endocrinol Metab (2014) 99(3):1072–80. doi: 10.1210/jc.2013-3978 24423366

[32] Naveh-Many T Volovelsky O. Parathyroid cell proliferation in secondary hyperparathyroidism of chronic kidney disease. Int J Mol Sci (2020) 21(12). doi: 10.3390/ijms21124332 PMC7352987 32570711

[33] Bilezikian JP Khan AA Silverberg SJ Fuleihan GE-H Marcocci C Minisola S. Evaluation and management of primary hyperparathyroidism: summary statement and guidelines from the fifth international workshop. J Bone Miner Res (2022) 37(11):2293–314. doi: 10.1002/jbmr.4677 36245251

[34] Sharma U Pal D. Prasad RJIjocb. *Alkaline phosphatase: an overview*. Indian J Clin Biochem (2014) 29(3):269–78. doi: 10.1007/s12291-013-0408-y PMC4062654 24966474

[35] Nizet A Cavalier E Stenvinkel P Haarhaus M Magnusson P. Bone alkaline phosphatase: An important biomarker in chronic kidney disease – mineral and bone disorder. Clinica Chimica Acta (2020) 501:198–206. doi: 10.1016/j.cca.2019.11.012 31734146

[36] Kamr AM Dembek KA Gilsenan W Bozorgmanesh R Hassan HY Rosol TJ. C-terminal telopeptide of type I collagen, osteocalcin, alkaline phosphatase, and parathyroid hormone in healthy and hospitalized foals. Domest Anim Endocrinology (2020) 72:106470. doi: 10.1016/j.domaniend.2020.106470 32408050

[37] Chen Z Zhang X Han F Xie X Hua Z Huang X. High alkaline phosphatase and low intact parathyroid hormone associate with worse clinical outcome in peritoneal dialysis patients. Peritoneal Dialysis Int (2020) 41(2):236–43. doi: 10.1177/0896860820918131 32363998

[38] Li J Molnar MZ Zaritsky JJ Sim JJ Streja E Kovesdy CP. Correlates of parathyroid hormone concentration in hemodialysis patients. Nephrol Dial Transplant (2013) 28(6):1516–25. doi: 10.1093/ndt/gfs598 PMC3685307 23348879

[39] Hamur H Kalkan K Duman H Durakoğlugil ME Küçüksu Z Inci S. Plateletcrit and platelet distribution width as independent predictors of coronary artery ectasia. Kosuyolu Heart J (2016) 19:173–8. doi: 10.5578/khj.20979

[40] Irkorucu O. Mean platelet volume and solitary parathyroid adenoma. Int J Clin Exp Med (2016) 9(7):14536–41. Available at: www.ijcem.com/ISSN:1940-5901/IJCEM0025401.

[41] Yilmaz H. Assessment of mean platelet volume (MPV) in primary hyperparathyroidism: effects of successful parathyroidectomy on MPV levels. Endocr Regul (2014) 48(4):182–8. doi: 10.4149/endo_2014_04_182 25512191

[42] Ren X Meng Z Liu M Zhu M He Q Zhang Q. No associations exist between mean platelet volume or platelet distribution width and thyroid function in Chinese. Medicine (2016) 95(40):e4573. doi: 10.1097/MD.0000000000004573 27749526 PMC5059028

[43] Ustuner B Basmaz S. PDW AS AN İNFLAMMATION MARKER IN PRIMARY HYPERPARATHYROİDİSM. Authorea (2021). doi: 10.22541/au.162798844.49257233/v1

[44] Rajan R Paul I Cherian KE Korula A Hephzibah J Manipadam MT. Myelofibrosis and pancytopenia associated with primary hyperparathyroidism. AACE Clin Case Rep (2021) 7(1):69–71. doi: 10.1016/j.aace.2020.11.018 33851024 PMC7924162

[45] Asghari G Yuzbashian E Wagner CL Mahdavi M Shamsi R Hosseinpanah F. The relation between circulating levels of vitamin D and parathyroid hormone in children and adolescents with overweight or obesity: Quest for a threshold. PloS One (2019) 14(11):e0225717. doi: 10.1371/journal.pone.0225717 31770397 PMC6879169

