# Supplementary material for: Assessing causal associations of hyperparathyroidism with blood counts and biochemical indicators: a Mendelian randomization study
Source: Front Endocrinol (Lausanne). 2023 Dec 11;14:1295040. doi: 10.3389/fendo.2023.1295040 (PMC10752421; doi:10.3389/fendo.2023.1295040)
Supplement: Supplementary Table 1 — Information of GWAS summary datasets used in MR analyses. [file DataSheet_1.docx]

**Supplementary Table 1.** Information of GWAS summary datasets used in MR analyses

| Traits | GWAS.ID |
| --- | --- |
| Hyperparathyroidism | [finn-b-E4_HYPERPARA](https://gwas.mrcieu.ac.uk/datasets/finn-b-E4_HYPERPARA/) |
| Alanine aminotransferase | ukb-d-30620_irnt |
| Albumin | ukb-d-30600_irnt |
| Alkaline phosphatase | ukb-d-30610_irnt |
| Apoliprotein A | ukb-d-30630_irnt |
| Apoliprotein B | ukb-d-30640_irnt |
| Aspartate aminotransferase | ukb-d-30650_irnt |
| Basophill percentage | ukb-d-30220_irnt |
| Calcium | ukb-d-30680_irnt |
| Cholesterol | ukb-d-30690_irnt |
| C-reactive protein | ukb-d-30710_irnt |
| Creatinine | ukb-d-30700_irnt |
| Cystatin C | ukb-d-30720_irnt |
| Direct bilirubin | ukb-d-30660_irnt |
| Eosinophill percentage | ukb-d-30210_irnt |
| Gamma glutamyltransferase | ukb-d-30730_irnt |
| Glucose | ukb-d-30740_irnt |
| Glycated haemoglobin | ukb-d-30750_irnt |
| Haematocrit percentage | ukb-d-30030_irnt |
| Haemoglobin concentration | ukb-d-30020_irnt |
| HDL cholesterol | ukb-d-30760_irnt |
| High light scatter reticulocyte count | ukb-d-30300_irnt |
| High light scatter reticulocyte percentage | ukb-d-30290_irnt |
| IGF-1 | ukb-d-30770_irnt |
| Immature reticulocyte fraction | ukb-d-30280_irnt |
| LDL direct | ukb-d-30780_irnt |
| Lipoprotein A | ukb-d-30790_irnt |
| Lymphocyte count | ukb-d-30120_irnt |
| Lymphocyte percentage | ukb-d-30180_irnt |
| Mean corpuscular haemoglobin | ukb-d-30050_irnt |
| Mean corpuscular haemoglobin concentration | ukb-d-30060_irnt |
| Mean corpuscular volume | ukb-d-30040_irnt |
| Mean platelet (thrombocyte) volume | ukb-d-30100_irnt |
| Mean reticulocyte volume | ukb-d-30260_irnt |
| Mean sphered cell volume | ukb-d-30270_irnt |
| Monocyte count | ukb-d-30130_irnt |
| Monocyte percentage | ukb-d-30190_irnt |
| Neutrophill count | ukb-d-30140_irnt |
| Neutrophill percentage | ukb-d-30200_irnt |
| Phosphate | ukb-d-30810_irnt |
| Platelet count | ukb-d-30080_irnt |
| Platelet crit | ukb-d-30090_irnt |
| Platelet distribution width | ukb-d-30110_irnt |
| Red blood cell (erythrocyte) count | ukb-d-30010_irnt |
| Red blood cell (erythrocyte) distribution width | ukb-d-30070_irnt |
| Reticulocyte count | ukb-d-30250_irnt |
| Reticulocyte percentage | ukb-d-30240_irnt |
| SHBG | ukb-d-30830_irnt |
| Testosterone | ukb-d-30850_irnt |
| Total bilirubin | ukb-d-30840_irnt |
| Total protein | ukb-d-30860_irnt |
| Triglycerides | ukb-d-30870_irnt |
| Urate | ukb-d-30880_irnt |
| Urea | ukb-d-30670_irnt |
| Vitamin D | ukb-d-30890_irnt |
| White blood cell (leukocyte) count | ukb-d-30000_irnt |
| Abbreviation: GWAS: genome-wide association study; ID: Identification; MR: Mendelian randomization; ukb: UK Biobank; HDL: High-density lipoprotein; IGF-1: Insulin-like growth factor 1; LDL: Low-density lipoprotein; SHBG: Sex hormone-binding globulin. | |

**Supplementary Table 2.** The F-statistics of IVs

| SNPs | F-Statistic |
| --- | --- |
| rs1104906 | 35.82 |
| rs184076217 | 86.81 |
| rs2383205 | 38.63 |
| rs3091842 | 31.73 |
| rs3843518 | 77.14 |
| rs56274948 | 29.93 |
| rs73186015 | 35.13 |
| rs9635741 | 33.26 |
| Abbreviation: IVs: instrumental variables; SNP: single nucleotide polymorphism. | |

**Supplementary Table 3.** MR analysis results

| Outcome | Method | Number of SNPs | Beta | 95% CI |
| --- | --- | --- | --- | --- |
| Alkaline phosphatase | IVW | 8 | 0.030 | 0.010~0.049 |
| Alkaline phosphatase | MR Egger | 8 | 0.050 | -0.033~0.133 |
| Alkaline phosphatase | WM | 8 | 0.027 | 0.008~0.046 |
| Calcium | IVW | 8 | 0.266 | 0.022~0.509 |
| Calcium | MR Egger | 8 | 0.318 | -0.740~1.375 |
| Calcium | WM | 8 | 0.080 | 0.049~0.111 |
| Mean platelet (thrombocyte) volume | IVW | 8 | 0.043 | 0.010~0.076 |
| Mean platelet (thrombocyte) volume | MR Egger | 8 | 0.077 | -0.063~0.217 |
| Mean platelet (thrombocyte) volume | WM | 8 | 0.030 | 0.007~0.052 |
| Phosphate | IVW | 8 | -0.114 | -0.214~-0.014 |
| Phosphate | MR Egger | 8 | -0.154 | -0.586~0.278 |
| Phosphate | WM | 8 | -0.035 | -0.066~-0.003 |
| Platelet count | IVW | 8 | -0.041 | -0.066~-0.016 |
| Platelet count | MR Egger | 8 | -0.005 | -0.109~0.098 |
| Platelet count | WM | 8 | -0.042 | -0.066~-0.019 |
| Platelet distribution width | IVW | 8 | 0.031 | 0.006~0.056 |
| Platelet distribution width | MR Egger | 8 | 0.072 | -0.031~0.176 |
| Platelet distribution width | WM | 8 | 0.022 | 0.002~0.043 |
| Vitamin D | IVW | 8 | -0.038 | -0.063~-0.013 |
| Vitamin D | MR Egger | 8 | 0.009 | -0.094~0.112 |
| Vitamin D | WM | 8 | -0.038 | -0.063~-0.012 |
| Abbreviation: MR: Mendelian randomization; SNP: single nucleotide polymorphism; CI: confidence interval; IVW: inverse-variance weighted; WM: Weighted median. | | | | |

**Supplementary Table 4.** Heterogeneity test results of associations with suggestive significant level

| Outcome | Q | Q_df | Q_*p* |
| --- | --- | --- | --- |
| Platelet count | 23.308 | 7 | 1.51E-03 |
| Alkaline phosphatase | 14.093 | 7 | 4.96E-02 |
| Vitamin D | 21.562 | 7 | 3.02E-03 |
| Mean platelet (thrombocyte) volume | 38.124 | 7 | 2.87E-06 |
| Platelet distribution width | 22.688 | 7 | 1.93E-03 |
| Phosphate | 337.900 | 7 | 4.79E-69 |
| Calcium | 1939.948 | 7 | 0.000 |

**Supplementary Table 5.** The pleiotropy results

| Outcome | Egger intercept | SE | *p*-value |
| --- | --- | --- | --- |
| Platelet count | -0.006 | 0.009 | 5.15E-01 |
| Alkaline phosphatase | -0.003 | 0.007 | 6.42E-01 |
| Vitamin D | -0.008 | 0.008 | 3.90E-01 |
| Mean platelet (thrombocyte) volume | -0.006 | 0.012 | 6.40E-01 |
| Platelet distribution width | -0.007 | 0.009 | 4.50E-01 |
| Phosphate | 0.007 | 0.036 | 8.58E-01 |
| Calcium | -0.009 | 0.087 | 9.24E-01 |
| Abbreviation: SE: standard error. | | | |

**Supplementary Table 6.** MR-PRESSO analyses for the results with identified outliers

| Outcome | MR Analysis | Causal Estimate | SD | *P*-value |
| --- | --- | --- | --- | --- |
| Platelet count | Outlier-corrected | -0.057 | 0.012 | 0.003 |
| Vitamin D | Outlier-corrected | -0.058 | 0.009 | 0.001 |
| Mean platelet (thrombocyte) volume | Outlier-corrected | 0.028 | 0.006 | 0.012 |
| Platelet distribution width | Outlier-corrected | 0.016 | 0.008 | 0.107 |
| Phosphate | Outlier-corrected | -0.122 | 0.026 | 0.019 |
| Calcium | Outlier-corrected | 0.259 | 0.008 | 0.001 |
| Alkaline phosphatase | Outlier-corrected | NA | NA | NA |
| Abbreviation: MR: Mendelian randomization; SD: standard deviation; NA: Not Applicable. | | | | |

| Exposure | Method | OR | 95%CI | *p*-value |
| --- | --- | --- | --- | --- |
| Alkaline phosphatase | IVW | 1.100 | 1.002~1.207 | 4.49E-02 |
| Alkaline phosphatase | WM | 1.156 | 0.999~1.337 | 5.23E-02 |
| Alkaline phosphatase | MR-Egger | 0.976 | 0.837~1.139 | 7.61E-01 |
| Calcium | IVW | 1.908 | 1.632~2.231 | 5.45E-16 |
| Calcium | WM | 1.986 | 1.611~2.449 | 1.33E-10 |
| Calcium | MR-Egger | 2.596 | 1.962~3.436 | 3.30E-10 |
| Mean platelet (thrombocyte) volume | IVW | 1.025 | 0.969~1.085 | 3.91E-01 |
| Mean platelet (thrombocyte) volume | WM | 0.981 | 0.885~1.088 | 7.19E-01 |
| Mean platelet (thrombocyte) volume | MR-Egger | 0.967 | 0.888~1.053 | 4.43E-01 |
| Phosphate | IVW | 0.747 | 0.612~0.912 | 4.13E-03 |
| Phosphate | WM | 0.859 | 0.688~1.072 | 1.78E-01 |
| Phosphate | MR-Egger | 0.731 | 0.525~1.017 | 6.52E-02 |
| Platelet count | IVW | 0.967 | 0.893~1.046 | 3.99E-01 |
| Platelet count | WM | 1.028 | 0.903~1.170 | 6.76E-01 |
| Platelet count | MR-Egger | 0.966 | 0.839~1.113 | 6.36E-01 |
| Platelet distribution width | IVW | 1.039 | 0.967~1.118 | 2.96E-01 |
| Platelet distribution width | WM | 1.012 | 0.889~1.152 | 8.58E-01 |
| Platelet distribution width | MR-Egger | 1.005 | 0.903~1.118 | 9.33E-01 |
| Vitamin D | IVW | 0.996 | 0.870~1.141 | 9.58E-01 |
| Vitamin D | WM | 0.952 | 0.773~1.172 | 6.43E-01 |
| Vitamin D | MR-Egger | 0.925 | 0.769~1.113 | 4.13E-01 |
| Abbreviation: MR: Mendelian randomization; SNP: single nucleotide polymorphism; OR: odds ratio; CI: confidence interval; IVW: inverse-variance weighted; WM: Weighted median. | | | | |

**Supplementary Table 7.** Bidirectional MR analyses results


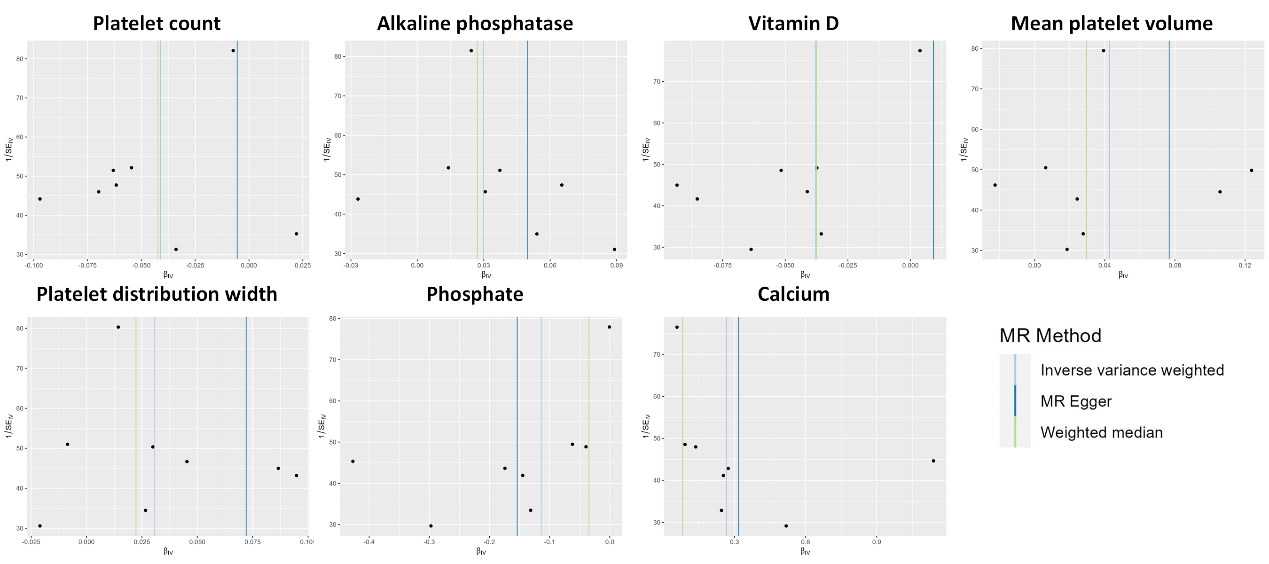


**Supplementary Figure 1.** Funnel plot indicating the causal associations of hyperparathyroidism on 7 blood counts and biochemical indicators, with each SNP acting as an IV. SNP: single nucleotide polymorphism; IV: instrumental variable; SE: standard error.
